# Epidermal growth factor receptor gene polymorphisms are associated with prognostic features of breast cancer

**DOI:** 10.1186/1471-2407-14-190

**Published:** 2014-03-14

**Authors:** Marcelo Sobral Leite, Letícia Carlos Giacomin, Diogo Nascimento Piranda, Juliana Simões Festa-Vasconcellos, Vanessa Indio-do-Brasil, Sérgio Koifman, Rodrigo Soares de Moura-Neto, Marcelo Alex de Carvalho, Rosane Vianna-Jorge

**Affiliations:** 1 Programa de Farmacologia, Coordenação de Pesquisa, Instituto Nacional do Câncer, Rua André Cavalcanti, 37, 3° andar CEP: 20231-050, Rio de Janeiro, RJ, Brazil; 2 Department of Molecular Pathology, Netherlands Cancer Institute, Plesmanlaan 121, 1066 CX, Amsterdam, Plesmanlaan 121; 3 Programa de Pós-Graduação em Farmacologia e Química Medicinal, Instituto de Ciências Biomédicas, Universidade Federal do Rio de Janeiro, Rio de Janeiro, RJ, Brazil; 4 Escola Nacional de Saúde Pública - FIOCRUZ, Rio de Janeiro, RJ, Brazil; 5 Departamento de Botânica, Instituto de Biologia, Universidade Federal do Rio de Janeiro, Rio de Janeiro, RJ, Brazil; 6 Instituto Nacional de Metrologia, Qualidade e Tecnologia, Rio de Janeiro, RJ, Brazil; 7 Instituto Federal do Rio de Janeiro, Rio de Janeiro, RJ, Brazil

**Keywords:** Breast cancer, EGFR, Gene polymorphisms, Gene expression, Prognostic estimates

## Abstract

**Background:**

The epidermal growth factor receptor (EGFR) is differently expressed in breast cancer, and its presence may favor cancer progression. We hypothesized that two *EGFR* functional polymorphisms, a (CA)n repeat in intron 1, and a single nucleotide polymorphism, *R497K*, may affect *EGFR* expression and breast cancer clinical profile.

**Methods:**

The study population consisted of 508 Brazilian women with unilateral breast cancer, and no distant metastases. Patients were genotyped for the *(CA)n* and *R497K* polymorphisms, and the associations between *(CA)n* polymorphism and EGFR transcript levels (n = 129), or between either polymorphism and histopathological features (n = 505) were evaluated. The REMARK criteria of tumor marker evaluation were followed.

**Results:**

(CA)n lengths ranged from 14 to 24 repeats, comprehending 11 alleles and 37 genotypes. The most frequent allele was *(CA)*_*16*_ (0.43; 95% CI = 0.40–0.46), which was set as the cut-off length to define the *Short* allele. Variant *(CA)n* genotypes had no significant effect in tumoral *EGFR* mRNA levels, but patients with two *(CA)n Long* alleles showed lower chances of being negative for progesterone receptor (OR_adjusted_ = 0.42; 95% CI = 0.19–0.91). The evaluation of *R497K* polymorphism indicated a frequency of 0.21 (95% CI = 0.19 – 0.24) for the variant (*Lys*) allele. Patients with variant *R497K* genotypes presented lower proportion of worse lymph node status (pN2 or pN3) when compared to the reference genotype *Arg/Arg* (OR_adjusted_ = 0.32; 95% CI = 0.17–0.59), which resulted in lower tumor staging (OR_adjusted_ = 0.34; 95% CI = 0.19-0.63), and lower estimated recurrence risk (OR = 0.50; 95% CI = 0.30-0.81). The combined presence of both *EGFR* polymorphisms (*Lys* allele of R497K and *Long/Long (CA)n*) resulted in lower TNM status (OR_adjusted_ = 0.22; 95% CI = 0.07-0.75) and lower ERR (OR = 0.25; 95% CI = 0.09-0.71). When tumors were stratified according to biological classification, the favorable effects of variant *EGFR* polymorphisms were preserved for luminal A tumors, but not for other subtypes.

**Conclusions:**

The data suggest that the presence of the variant forms of *EGFR* polymorphisms may lead to better prognosis in breast cancer, especially in patients with luminal A tumors.

## Background

Breast cancer is the most frequent type of cancer in women both in the developed and the developing world [1]. It is a very heterogeneous disease with regards to its molecular profile [2], and clinical course, which presents great interpatient variability. Although conventional histopathological characteristics remain the most important prognostic determinants of survival [3], there is a continuous search for new biomarkers or stage models that could help predicting clinical evolution [4], or improving therapy selection. In this regard, genetic variations in carcinogenesis-related processes are natural candidates for exploring new prognostic factors or potential targets for specific therapies [5,6].

The epidermal growth factor receptor (EGFR) is a transmembrane tyrosine kinase (TK) receptor of the ErbB family, whose activation leads to mitogenic signaling [7]. EGFR is frequently overexpressed in many tumors, including breast cancer, and its activation contributes to unrestricted proliferation, advanced stages of disease, resistance to conventional treatments, and poor prognosis [8]. Despite the recognition that EGFR overexpression in breast tumors may affect disease progression [8], the responses of anti-EGFR therapies in breast cancer are not fully satisfactory [9], and the reasons for this clinical variability are not fully understood.

The *EGFR* gene, located at 7p12.3-p.1, contains multiple polymorphisms [10], two of which are recognized for their functional effects: a dinucleotide (CA)n repeat sequence polymorphism in intron 1 (rs72554020) affects gene transcription [11], and appears to modulate EGFR expression in breast tumors [12], and a single nucleotide change (G → A) in exon 13 leads to an Arginine (Arg) → Lysine (Lys) substitution in codon 497 (rs11543848), resulting in attenuated TK activity, with consequent reductions in ligand binding, growth stimulation, and induction of proto-oncogenes *myc*, *fos*, and *jun*[13].

In the present work, we aimed to describe the frequency of these two *EGFR* polymorphisms among Brazilian breast cancer patients, and to evaluate their impact on breast cancer prognosis, exploring the effects of *(CA)n* polymorphism on EGFR transcript levels, and the associations of both polymorphisms with histopathological features and prognostic estimates.

## Materials and methods

### Subjects and study design

The study population consisted of a prospective cohort of Brazilian women with first diagnosis of unilateral breast cancer and no distant metastases, admitted at the Brazilian National Cancer Institute (INCA) during the period from February 2009 to April 2011, and who were assigned for tumor resection as their first therapeutic approach. The recruitment occurred before surgery, but the inclusion was only completed after diagnosis confirmation by histopathological evaluation of the resected tumor. The study protocol was approved by the Ethics Committee of the Brazilian National Cancer Institute (INCA #129/08), and all patients gave written consent to participate. The REMARK guidelines (REporting recommendations for tumor MARKer prognostic studies) were followed [14].

### Histopathological characterization

The histopathological evaluation of resected tumors was performed following institutional routine procedures, and all individual data were obtained from electronic medical records. The histopathological characterization was based on the TNM classification by the American Joint Committee on Cancer [15] and on the Elston Ellis histological grading system [16].

The data on hormone receptors, i.e. Estrogen Receptor (ER), and Progesterone Receptor (PR), and on the Human Epidermal growth factor Receptor 2 (HER2) status were used for biological classification of the tumors, as proposed by Huober *et al*. [17]. The Estimated Recurrence Risk (ERR) was inferred by a combination of all histopathological features, as proposed by the Early Breast Cancer Trialists’ Collaborative Group [18], with the following categories: “Low Risk”, characterized by the presence of [age ≥ 35 years, N0 (absence of tumor cells in lymph nodes), G1 (histological grade 1), T1 (tumor size lower than 2 cm), (ER+ or PR+), HER2-], and absence of peritumoral vascular invasion; “Intermediate Risk”, characterized by N0 in the presence of [age < 35 years, or T ≥ 2, or G ≥ 2, or (ER- and PR-), or HER2+], or by N1 (presence of tumor cells in 1 to 3 lymph nodes) in the presence of [HER2-, and (ER + or PR+)]; and “High Risk”, characterized by N1 in the presence of [HER2+, or (ER- and PR-)], or by N ≥ 2 (presence of tumor cells in more than 3 lymph nodes).

### Genotyping analyses

Peripheral blood samples (3 mL) were collected from the subjects, and DNA was extracted using the Blood Genomic Prep Mini Spin Kit (GE Heathcare, Buckinghamshire, UK), following the procedures recommended by the manufacturer. The genotyping analyses were performed using PCR-RFLP for the SNP *R497K* (rs11543848) or by capillary electrophoresis for the (CA)n repeat polymorphism in intron 1 (rs72554020). The PCR amplifications were performed with the following primers (Life Thechnology, Carlsbad, CA, USA): 5′-AGGTCTGCCATGCCTTGT-3′ (sense) and 5′-CAACGCAAGGGGATTAAAGA-3′ (antisense) for *R497K*; or 5′-TTCTCCTCAAAACCCGGAGAC-3′ labeled with 6-FAM™ (sense) and 5′-GTCACGAAGCCAGACTCGCT-3′ for (CA)n repeat (antisense). The *R497K* PCR products (5 μL) were digested with 5U of *Bst*N1 restriction enzyme (New England BioLabs, Northbrook, IL, USA) at 60°C for 3 hours, and the digestion products were resolved on 2% agarose gel and stained with ethidium bromide for visualization under UV light. The digestion of the homozygous G alleles (Arginine) produced two fragments (100 bp and 56 bp), whereas the homozygous A alleles (Lysine) remained intact (156 bp). The method was validated by direct sequencing of four samples of each genotype.

The (CA)n repeat PCR products (0.5 μl) were denatured at 95°C for 3 min in the presence of 0.5 μl of the GeneScan™ 400HD ROX molecular weight standard (Applied Biosystems, Foster City, CA, USA) and 9.0 μl of Hi-Di™ Formamide (Life Thechnology, Carlsbad, CA, USA), refrigerated to 4°C for 2 min, and then submitted to separation by capillary electrophoresis in ABI Prism® 3130 Genetic Analyzer, using POP7™ polymer (Applied Biosystems, Foster City, CA, USA). The analyses were performed using the GeneMapper® Software v.3.7 (Applied Biosystems, Foster City, CA, USA). The PCR products identified as homozygous, i.e. those presenting a single retention time at the capillary electrophoresis, were submitted to direct sequencing, using the BigDye® Terminator Kit (Applied Biosystems, Foster City, CA, USA), in order to establish a correspondence between each retention time and the respective number of CA repeats (or allele length).

### Quantification of EGFR mRNA

Fresh specimens of breast tumors were dissected by clinical pathologists after tumor resection, frozen in liquid N_2_, and stored at the Brazilian National Bank of Tumors (BNT-INCA). Frozen sections of breast specimens (with approximately 2 mm) were used for RNA isolation, which was performed using the RNeasy Mini Kit (Qiagen, Valencia, CA, USA), following the manufacturer’s instructions. The RNA samples were stored in RNAse-free distilled water at -80°C, and the corresponding cDNA was synthesized using 2 μg of RNA, with High Capacity cDNA Reverse Transcription Kit (Applied Biosystems, Foster City, CA, USA), according to the manufacturer’s instructions.

The relative quantification of *EGFR* transcripts was performed using quantitative real-time RT-PCR (TaqMan) assays, in an ABI PRISM 7500 Sequence Detector System (Applied Biosystems, Foster City, CA, USA). Each reaction contained: cDNA templates (approximately 40 ng), 10 μl of reaction mix containing 5 μl Taqman® Gene Expression Master Mix, and Taqman® probes, which were as follows: *EGFR* Hs01076078_m1 (with FAM), *PPIA* 4326316E (with VIC) (Applied Biosystems, Foster City, CA, USA). The thermal cycling conditions comprised an initial denaturation step at 95°C for 10 min, followed by 40 cycles of 95°C denaturation for 15 sec, and annealing at 60°C for 1 min. The experiments were carried out in 96-well plates, including a nontemplate control, and a reference control, consisting of cDNA obtained from a commercial Human Mammary Gland (HMG) total RNA (Clontech Laboratories, Mountain View, CA, USA). The relative quantification of *EGFR* mRNA was calculated as the average 2^-ΔΔCt^, where ΔΔCT = ΔCT_*EGFR*_ - ΔCT_HMG,_ and ΔCT_*EGFR*_ = Ct_*EGFR*_ - Ct_*PPIA*_*,* and ΔCT_*HMG*_ = Ct_*HMG*_ - Ct_*PPIA*_. All data were generated in triplicates and expressed as median +/- SD with the 25–75 percentiles.

### Statistical analyses

A descriptive study of the cohort was conducted, presenting measures of central tendency and dispersion for continuous variables, or relative frequencies for each categorical variable. Allelic and genotypic frequencies were derived by gene counting. The histopathological features were dichotomized for better and worse prognostic values, and their associations with *EGFR* genotypes were evaluated by the *Chi*-square or Fisher’s exact tests. In the cases of significant associations between *EGFR* genotypes and independent histopathological variables, the odds ratios (OR) and their respective 95% confidence intervals (95% CI) were tested for linear-by-linear associations, with calculation of trend significances (P_trend_), and definition of phenotypic inheritance models. The odds ratios between *EGFR* phenotypic groups and histopathological categorical features were adjusted for all other independent clinical variables (OR_adjusted_) using multiple regression analyses. The comparison of the relative quantities of *EGFR* mRNA as a function of histopathological features or *EGFR* genotypes was performed with the GraphPad Prism 5.0 software (GraphPad Software, La Jolla, CA, USA), using the non-parametric Mann–Whitney *U*-test for comparison of two groups, or the Kruskal-Wallis test for comparison of multiple groups. All other statistical analyses were conducted using SPSS 13.0 for Windows (SPSS Inc., Chicago, Illinois). The threshold for significance was set at P < 0.05.

## Results

### Characterization of the cohort

A total of 576 patients were recruited when admitted for surgery, and 528 had the diagnosis and inclusion criteria confirmed after pathological evaluation of their resected tumor. Blood samples were available for 511 cases, and 508 of them had good DNA quality for genotyping assays. Table 1 presents the main clinical and histopathological characteristics, as well as the genotypic distribution of *EGFR* polymorphisms for the 508 patients evaluated. The median age was 59 years old, ranging from 27 to 92.

**Table 1:** **Histopathological characteristics, tumor classification and *EGFR *** **genotypes in Brazilian breast cancer patients**

**Independent variables**	**n**	**%**	**Tumor classifications**	**n**	**%**
Histological type					
Ductal invasive	440	8606	0	35	7.1
Ductal *in situ*	34	6.7	IA	157	31.7
Lobular *in situ*	1	0.2	IB	7	1.4
Lobular invasive	29	5.7	II A	148	29.9
Mixed	4	0.8	II B	70	14.1
			III A	52	10.5
Tumor grade			III B	1	0.2
Grade 1	60	12.6	III C	25	5.1
Grade 2	193	40.5	missing	13	
Grade 3	223	46.8			
Missing	32		Biological classifiation		
			luminal A	293	64.8
Tumor size			luminal B	89	5.8
pTis	35	7.1	HER2 like	26	5.8
pT1	226	45.7	triple negative	44	9.7
pT2	220	44.4	missing	56	
pT3	13	2.6			
pT4	1	0.2	ERR categories		
Missing	13		low	20	4.2
			intermediate	357	75.5
Lymph node status			high	96	20.3
pN0	312	61.4	missing	35	
pN1	120	23.6			
pN2	50	9.8			
pN3	26	5.1			
Estrogen receptor			**EGFR Genotypes**	***n***	**%**
Negative	76	15.0	*R497K*		
Positive	414	84.5	*Arg/arg*	308	61.0
Missing	18		*Arg/Lys*	176	34.9
			*Lys/Lys*	21	4.2
Progesterone receptor			missing	3	
Negative	134	26.4			
Positive	355	72.6			
Missing	19				
			*(CA)n*		
HER2			*Short/Short*	108	22.6
Negative	378	74.4	*Short/Long*	230	48.2
Indeterminate	15	3.2	*Long/Long*	139	29.1
Positive	73	15.7	missing	31	
Missing	42				

Data are expressed as the number of patients and their respective percentage in each category. *(CA)n Short*: n≤ 16; *(CA)n Long*: n > 16. *Abbreviations*: *ERR* Estimated Recurrence Risk, *HER2* Human Epidermal growth factor Receptor 2.

### Characterization of *EGFR* polymorphisms

The genotyping of the *R497K* polymorphism was obtained for 505 patients, whereas the characterization of the number of (CA)n repeats by electrophoresis was conclusive in 477 cases (Table 1). The frequency of the variant *R497K* allele (*Lys*) was 0.21 (95% CI = 0.19-0.24).

**Figure 1:**
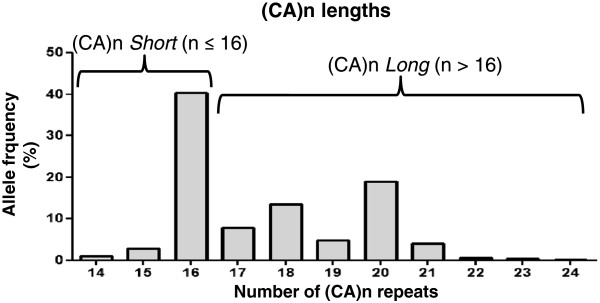
**Evaluation of the (CA)n lengths in the Brazilian cohort of breast cancer patients: (CA)n ≤ 16 were grouped as *Short *** **alleles, whereas (CA)n > 16 were grouped as *Long *** **alleles.**

The evaluation of the (CA)n lengths indicated a range of 14 to 24 repeats (Figure 1), comprehending 11 alleles and 37 genotypes. The most frequent allele was *(CA)*_*16*_ (0.43; 95% CI = 0.40–0.46), which was taken as the cut-off length to group *Short* alleles. All the other variant alleles, with more than 16 (CA) repeats were considered as *Long* alleles. Thus, the genotypic distribution used for further analyses was: *Short/Short* (reference homozygous genotype), S*hort/Long* (heterozygous) and *Long/Long* (variant homozygous genotype).

### Characterization of EGFR mRNA expression in breast tumors, and evaluation of the influence of *(CA)n* genotypes and of histopathological characteristics

The *EGFR* mRNA expression levels were evaluated in fresh-frozen tumor samples from 129 patients. Table 2 shows the main clinical and histopathological characteristics, as well as the genotypic distribution of *EGFR* polymorphisms in this subcohort. The data are presented in comparison with those described for the general population (Table 1). The results indicate that the subcohort whose tumors were used for expression analyses is similar to the general population, except for tumor size, and nodal status, which tend to be higher in the former. This difference is caused by the institutional biobank policy, which restricts collection of tumors with less than 1 cm for non-diagnostic purposes.

**Table 2:** Characterization of a subcohort used for tumoral RNA analyses, and comparison with the complete cohort

	**Complete cohort**		**Tumor subcohort**		
**Individual variables**	**Total**	**n = 508**	**Total**	**n = 129**	**P**
	**n**	**%**	**n**	**%**	
Age					
> 35 years-old	496	97.6	125	96.9	0.63
≤ 35 years-old	12	2.4	4	3.1	
Family history					
No	400	87.7	85	85.9	0.61
Yes	56	12.3	14	14.1	
Histological type					
*in situ*	35	7.0	2	1.6	**0.018**
Invasive	462	93.0	127	98.4	
Tumor grade					
Grade 1	60	12.6	10	7.8	0.14
Grade 2 or 3	416	87.4	117	90.7	
Tumor size					
pT ≤ 2 cm	226	49.1	42	33.1	**0.001**
pT > 2 cm	234	50.9	85	66.9	
Lymph node status					
pN0 or pN1	432	85.0	99	76.7	**0.024**
pN2 or pN3	76	15.0	30	23.3	
ER/PR status					
Negative (both)	76	15.5	27	20.9	0.14
Positive (either)	414	84.5	102	79.1	
HER2 status					
Negative	378	83.8	102	81.0	0.45
Positive	73	16.2	24	19.0	
Biological classification					
Non triple negative	440	90.9	112	86.8	0.17
Triple negative	44	9.1	17	13.2	
*EGFR* genotypes					
*R497K*					
*Arg/Arg*	308	61.0	82	64.1	0.380
*Arg/Lys*	176	34.9	38	29.7	
*Lys/Lys*	21	4.2	8	6.3	
*(CA)n*					
*Short/Short*	108	22.6	30	23.6	0.358
*Short/Long*	230	48.2	68	53.5	
*Long/Long*	139	29.1	29	22.8	

*Abbreviations*: *ER* Estrogen receptor, *PR* Progesterone receptor, *HER2* Human Epidermal groth factor Receptor 2. Statistically significant differences are presented in bold characters.

**Figure 2:**
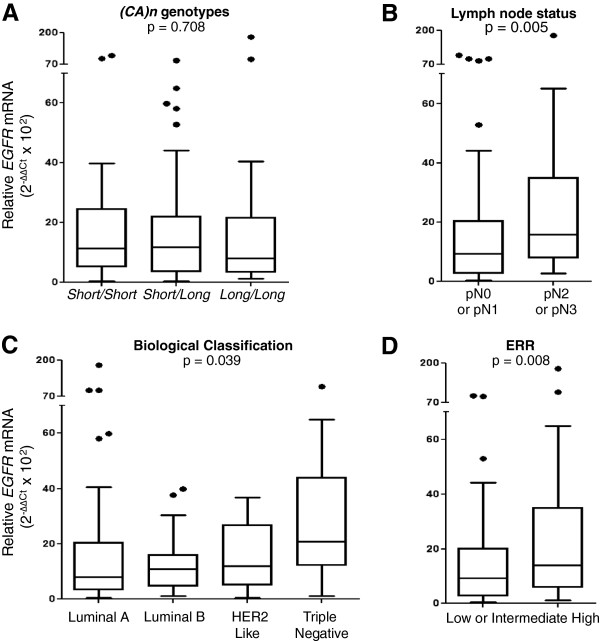
**Expression levels of *EGFR *mRNA in breast tumors according to *EGFR *genotypes and histopathological prognostic estimates: (A)***(CA)n* genotypes, presented as *Short/Short*, (n = 30) *Short/Long* (n = 68) or *Long/Long* (n = 29); **(B)** Lymph node status, presented as pN0 or pN1 (n = 99) or pN2 or pN3 (n = 30); **(C)** Biological classification of tumors, presented as Luminal A (n = 69), Luminal B (n = 30), HER2 like (n = 10) or triple negative (n = 17); **(D)** Estimated Recurrence Risk (ERR), divided in “Low or intermediate” (n = 50) and “High” (n = 50).

The relationship between *EGFR* mRNA expression levels and *(CA)n* genotypes or prognostic categories of breast tumors were explored (Figure 2). The results indicate no differences related to *(CA)n* genotypes (Figure 2A), whereas the lymph node status (Figure 2B) and the biological subclassification (Figure 2C) showed significant influences. The *EGFR* mRNA expression levels were significantly higher for patients with worse lymph node status, as well as for triple-negative tumors when compared to all other subgroup classifications (p = 0.003). As a consequence of these two associations, patients with higher ERR presented higher *EGFR* mRNA expression levels (Figure 2D).

### Association between *EGFR* genotypes and prognostic variables

Table 3 presents the distribution of *R497K* and *(CA)n* genotypes according to prognostic categories. The distribution of *R497K* genotypes was statistically different as a function of the lymph node status, whereas the distribution of *(CA)n* genotypes was statistically different as a function of the PR status.

**Table 3:** **Distribution of the *R497K *** **and *(CA)n *** **genotypes according to prognostic variables**

**Prognostic variables**		***R497K***					***(CA)n***			
		***Arg/Arg***	***Arg/Lys***	***Lys/Lys***			***Short/short***	***Short/long***	***Long/long***	
	**Total**	**n = 308**	**n = 176**	**n = 21**		**Total**	**n = 108**	**n = 230**	**n = 139**	
	**n**	**%**	**%**	**%**	**P**	**n**	**%**	**%**	**%**	**P**
Age										
> 35 years-old	493	61.7	34.1	4.3	0.06	466	22.7	48.5	28.8	0.48
≤ 35 years-old	112	33.3	66.7	0.0		11	18.2	36.4	45.5	
Familial history										
No	400	60.0	36.0	4.0	0.99	376	21,5	49,7	28,7	0.38
Yes	56	60.7	35.7	3.6		53	26,4	39,6	34.0	
Histological type										
*in situ*	35	62.9	34.3	2.9	0.91	33	15.2	48.5	36.4	0.44
Invasive	459	61.0	34.6	4.4		434	23.3	48.6	28.1	
Tumor grade										
Grade 1	60	56.7	40.0	3.3	0.64	58	25.9	46.6	27.6	0.74
Grade 2 or 3	413	61.3	34.1	4.6		389	21.3	48.8	29.8	
Tumor size										
pT ≤ 2 cm	224	59.4	36.6	4.0	0.65	214	22.9	45.3	31.8	0.24
pT > 2 cm	233	62.7	32.6	4.7		218	23.4	51.8	29.8	
Lymph node status										
pN0 or pN1	429	58.0	37.3	4.7	**0.005**	405	22.7	47.2	30.1	0.47
pN2 or pN3	76	77.6	21.1	1.3		72	22.2	54.2	23.6	
ER status										
Negative	76	68.4	27.6	3.9	0.38	72	26.4	51.4	22.2	0.29
Positive	411	60.1	35.5	4.4		387	21.2	47.8	31.0	
PR status										
Negative	133	63.2	32.3	4.5	0.87	128	28.1	50.0	21.9	**0.031**
Positive	353	60.9	34.8	4.2		330	19.4	47.9	32.7	
HER2 status										
Negative	376	59.3	35.6	5.1	0.13	356	21.9	50.0	28.1	0.30
Positive	72	65.3	34.7	0.0		67	20.9	41.8	37.3	
Biological classification										
Luminal A	291	58.8	36.4	4.8	0.68	275	20.0	49.8	30.2	0.35
Luminal B	88	60.2	36.4	3.4		83	21.7	43.4	34.9	
HER2 like	26	73.1	26.9	0.0		24	37.5	41.7	20.8	
Triple negative	44	63.6	29.5	6.8		42	23.8	54.8	21.4	

Data are expressed as the number of patients and respective percentages in each category. *(CA)n Short*: n ≤ 16; *(CA)n Long*: n > 16. *Abbreviations*: *ER* Estrogen Receptor, *PR* Progesterone Receptor, *HEr2* Human Epidermal groth factor Receptor 2. Statistically significant differences are presented in bold characters.

**Figure 3:**
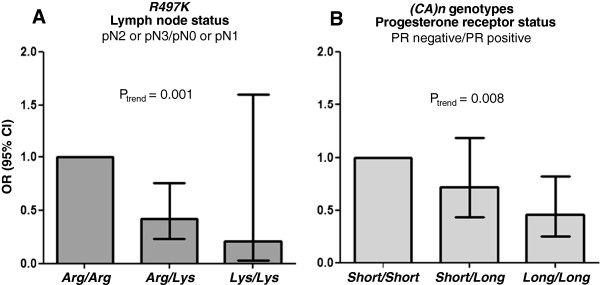
**Significant influences of *EGFR *polymorphisms on histopathological features of brast cancer: (A)** Proportion of lymph node status (pN2 or pN3/pN0 or pN1) according to *R497K* genotypic groups; **(B)** proportion of progesterone receptor status (PR negative/PR positive) according to *(CA)n* genotypic groups.

The association between *R497K* genotypes and lymph node status, or between *(CA)n* genotypes and the PR status is further explored in Figure 3. Figure 3A shows that patients with the heterozygous genotype *Arg/Lys* presented lower proportion of the worse lymph node status (pN2 or pN3), when compared to the reference homozygous genotype *Arg/Arg* (OR = 0.42; 95% CI = 0.23–0.76), whereas among patients with the homozygous variant genotype *Lys/Lys* (n = 21), there was only 1 case of pN2 or pN3 (OR = 0.21; 95% CI = 0.028–1.60). These results indicate that the magnitude of the association between *R497K* polymorphism and lymph node status depends on the number of variant *Lys* alleles (P_trend_ = 0.001). Similarly, Figure 3B shows the impact of the number of variant *(CA)n* alleles on the proportion of negative PR status. The results indicate an apparently progressive effect of the number of long *(CA)n* alleles (P_trend_ = 0.008). Thus, patients with the *Short/Long* genotype showed a slightly lower proportion of negative PR status when compared to the reference *Short/Short* genotype (OR = 0.72; 95% CI = 0.44–1.19), and a significant protective effect was observed for the variant *Long/Long* genotype (OR = 0.46; 95% CI = 0.26–0.83).

### Interaction between *EGFR* polymorphisms

The above trend analyses suggested an inheritance model of codominance for the association between *R497K* polymorphism and lymph node status and of recessiveness for *(CA)n Long* allele and PR status. Thus, the genotypes *Arg/Lys* and *Lys/Lys* were grouped for evaluation of their impact on lymph node status, whereas the *(CA)n Long/Long* genotype was evaluated in comparison with the combined *Short/Short* and *Short/Long* genotypes for its effect on the PR status. The two *EGFR* polymorphisms were also evaluated in a combined analysis in order to investigate a possible interaction between them on the distribution of breast cancer prognostic features (Table 4).

**Table 4:** Impact of EGFR polymorphisms on histopathological features and prognostic estimates of breast cancer

	***R497K *** **variant genotypes**			***(CA)n *** **variant genotypes**			***EGFR *** **combined variant genotypes**	
	**Lymph node status**	**TNM status**	**ERR**	**PR status**	**TNM status**	**ERR**	**TNM status**	**ERR**
**Tumor stratification**	**N2/N3**	**≥ IIIA**	**High**	**negative**	**≥ IIIA**	**High**	**≥ IIIA**	**High**
	**OR adjusted**	**OR adjusted**	**OR**	**OR adjusted**	**OR adjusted**	**OR**	**OR adjusted**	**OR**
	**(IC95%)**	**(IC95%)**	**(IC95%)**	**(IC95%)**	**(IC95%)**	**(IC95%)**	**(IC95%)**	**(IC95%)**
	**P**	**P**	**P**	**P**	**P**	**P**	**P**	**P**
Complete cohort	0.32	0.34	0.50	0.42	0.74	0.74	0.22	0.25
	(0.17-0.59)	(0.19-0.63)	(0.30-0.81)	(0.19-0.91)	(0.40-1.32)	(0.43-1.25)	(0.07-0.75)	(0.09-0.71)
	**< 0.001**	**0.001**	**0.005**	**0.030**	0.53	0.26	**0.018**	**0.005**
Luminal A	0.34	0.38	0.35	NA	0.62	0.60	0.32	0.24
	(0.15-0.75)	(0.18-0.80)	(0.17-0.75)		(0.29-1.36)	(0.27-1.32)	(0.092-1.12)	(0.06-1.03)
	**0.007**	**0.011**	**0.005**		0.22	0.20	0.075	**0.038**
Luminal B	0.057	0.060	0.58	0.080	1.29	1.29	NC	0.61
	(0.006-0.53)	(0.007-0.53)	(0.21-1.60)	(0.012-0.54)	(0.35-4.72)	(0.48-3.48)		(0.12-3.10)
	**0.012**	**0.012**	0.29	**< 0.001**	0.26	0.62		0.54
HER2-like	0.18	0.22	0.27	NA	1.14	2.91	NC	NC
	(0.02-2.02)	(0.02-2.28)	(0.05-1.64)		(0.15-8.59)	(0.27-31.21)		
	0.18	0.22	0.14		0.90	0.36		
Triple negative	5.2	5.1	2.0	NA	NC	NC	NC	NC
	(0.48-55.27)	(0.48-54.03)	(0.48-8.40)					
	0.17	0.18	0.349					

*R497K* variant genotypes include *Arg/Lys* and *Lys/Lys.* The genotype *Arg*/*Arg* was taken as reference for OR calculation*; (CA)n* variant genotypes include two *Long* alleles in combination (*Long/Long)*, where the *Long* allele corresponds to (CA)n lengths with more than 16 repeats. The genotypes *Short/Short* and *Short/Long* were considered together as reference for OR calculation. *EGFR* combined variants include patients with genotypes *Arg/Lys* or *Lys/Lys* for *R497K* and *Long/Long* for *(CA)n.* The reference genotypes were *R497K Arg/Arg* and *(CA)n Short/Shor*t or *Short/Long.* The OR calculation for lymph node status was adjusted for age, hystological type, tumor grade, tumor size, and for ER, PR and HER2 status. The OR calculation for PR status was adjusted for age, hystological type, tumor grade, tumor size, lymph node status and for ER and HER2 status. The OR calculation for TNM status was adjusted for age, hystological type, tumor grade and for ER, PR and HER2 status. Statistically significant differences are presented in bold characters. *Abbreviations*: *TNM* Tumor staging according to tumor size, lymph node status and distant metastases, *ERR* Estimated Recurrence Risk, *PR* Progesterone Receptor, *HER2* Human Epidermal growth factor Recetor 2, *NA* not applicable, *NC* not calculated (the number of samples was not sufficient for OR calculation).

The results indicate a significantly protective effect of the *Lys* allele on the proportion of the worse lymph node status after adjustment for other independent individual prognostic variables. As a consequence, patients carrying the *Lys* allele showed lower TNM status and lower ERR. With regards to the *(CA)n* polymorphism, the association between *(CA)n Long/Long* genotype and PR negative status also remained significant after adjustment for other independent individual prognostic variables (OR_adjusted_ = 0.42; 95% CI = 0.19-0.91), but did not affect TNM status or the ERR. When the two *EGFR* polymorphisms are present, there is lower TNM status (OR_adjusted_ = 0.22; 95% CI = 0.07-0.75) and lower ERR (OR = 0.25; 95% CI = 0.09-0.71).

The stratification of breast tumors according to their biological classification indicates that the association between combined variant *EGFR* polymorphisms and better lymph node status occurs for tumors classified as luminal A, but not for the other biological subtypes.

## Discussion

The distribution of the two *EGFR* functional polymorphisms in the Brazilian population was not known before the current study. Our data indicate a frequency of 0.21 (95% CI = 0.19 – 0.24) for the *497 K* (*Lys* allele), and of 0.43 (95% CI = 0.40 – 0.46) for the *(CA)*_*16*_. These results are similar to the frequencies reported for Europeans and North-Americans (including African-Americans), either for *R497K* polymorphisms [19,20] or *(CA)n*[12,21]. Asian populations, however, appear to have higher frequencies of the *Lys* allele [22,23], and different patterns of *(CA)n* alleles [12,21,24,25].

One difficulty of evaluating the effects of *(CA)n* polymorphism in gene transcriptional activity *in vivo* is the vast distribution of the number of (CA) repeats, with various possible heterozygous genotypes, and no clear model on how the two alleles interact for the final cell phenotype. Amador *et al*. [26] considered the sum of CA repeats of both alleles and showed an inverse correlation between this combined length and the levels of *EGFR* mRNA in head and neck cancer cell lines. Buerger *et al*. [12], studying breast tumors, considered the length of the smaller allele, and showed a non-significant tendency for lower EGFR protein expression with increasing allele length. Accordingly, Buerger *et al*. [27] showed that breast tumors from Japanese patients, who present high frequencies of *(CA)*_*20*_ and other long alleles, had lower amounts of EGFR protein than tumors from German patients, who have a predominance of *(CA)*_*16*_ and other short alleles. Other authors, however, found no correlation between the length of the (CA)n region and the relative quantification of *EGFR* mRNA [28] or EGFR protein expression [29].

Our data confirm the great dispersion of (CA) lengths and indicate great variability on the expression of *EGFR* mRNA, with no apparent inverse correlation between the number of (CA) repeats, considering either the smaller allele or the combined length within each genotype (data not shown). In order to investigate a possible effect of somatic mutations on the tumoral *(CA)n* genotype, we evaluated a set of 40 tumor samples. The number of CA repeats was preserved in relation to genomic DNA in all cases (data not shown). Although we did not extend such analyses to all patients, it appears that mutational events, such as loss of heterozigosity, are not affecting the *EGFR* locus of breast tumors. Nevertheless, an accurate characterization of the impact of *EGFR* polymorphisms on the gene transcriptional activity *in vivo* would ideally include quantification of gene amplification in the tumors [27]. In addition, there are two other *EGFR* polymorphisms (-*216G/T* or rs712829 and *-191C/A* or rs712830), located in the promoter region, which might have functional impact on *EGFR* transcriptional activity [30]. Finally, epigenetic variations may also interfere with *EGFR* expression [31].

The evaluation of the impact of *EGFR* polymorphisms on histopathological and molecular characteristics of breast cancer indicated significant association between *R497K* variant genotypes and better lymph node status, corroborating the findings of Kallel *et al*. [32], and between *Long*/*Long (CA)n* genotypes and positive PR status. These two associations seem protective in relation to breast cancer evolution, since a greater number of affected lymph nodes increases the risk of systemic metastasis [33], and the lack of PR expression increases the risk of disease progression, especially in post-menopausal women [34].

With regards to the molecular mechanisms underlying lymph node metastases, EGFR appears to activate integrins [35] and metaloproteinases [36], favoring cell differentiation towards an invasive phenotype. The association between the variant allele (*Lys*) and better lymph node status appear to corroborate the notion of reduced signaling with the variant EGFR isoform [13], leading to lower invasiveness, which reinforce the role of EGFR in breast cancer pathogenesis.

The interaction between the EGFR activity and the PR status might occur via a cross-talk mechanism between steroid and growth factor receptors [37], resulting in activation of the PIK3-Akt-mTOR pathway, which appears to negatively modulate the transcriptional activity of the PR [38]. This negative modulation of ER-mediated functions in breast cancer via EGFR signaling may underlie the mechanism of resistance to hormone therapy observed in tumors with high EGFR expression [39]. Taken together, the association between *EGFR* polymorphisms and lymph node metastases and negative PR status appear to corroborate the role of EGFR in breast cancer pathogenesis.

The combined presence of *Long/Long (CA)n* genotypes and *Lys R497K* alleles appears to favor better prognostic estimates in breast cancer. Other studies involving different types of cancer also point to an interaction between the two *EGFR* polymorphisms, with a combined protective effect in relation to disease progression. Zhang *et al*. [40], evaluating pelvic recurrence in patients with rectal cancer treated with chemoradiation, showed that the highest risk for local recurrence was seen in patients with the reference genotypes, i.e., both 497 *Arg* alleles and <20 CA repeats. Bandrés *et al*. [41], studying head and neck cancer, showed that patients with at least one 497 *Arg* allele and both (CA)n repeats ≤ 16 presented higher risk of death. Press *et al*. [42], studying metastatic colon cancer, found that men with the *Arg/Arg* genotype and two short alleles (< 20 CA repeats) had shorter overall survival than men with the *Lys/Lys* or *Arg/Lys* variant genotypes and any long allele (≥ 20 CA repeats).

The stratification of breast tumors according to their biological subtypes suggests that the apparently protective effects of *EGFR* polymorphisms are characteristic of luminal A tumors. This apparently selective effect of *EGFR* polymorphisms might be due to the lower genomic instability of luminal A tumors in relation to other subtypes, which present more aggressive phenotypes due to superposed molecular alterations [43]. Nevertheless, the small number of non-luminal A tumors limits the statistical power of the analyses, and the confidence of this assumption. In addition, the apparently favorable associations of *EGFR* polymorphisms with prognostic features at diagnosis cannot be considered as actually predictive of disease progression or therapy response,

## Conclusions

In conclusion, the current results indicate a potential benefit of *EGFR* polymorphisms as independent prognostic factors, especially in early-stage luminal A tumors, as they might contribute to identify patients at higher risk of progression. We propose that *EGFR* genotyping should be further evaluated for their prognostic value in prospective studies of breast cancer survival.

### Ethical standards

The study was conducted following the international precepts of ethics in research and of good clinical practice. The authors complied with the Brazilian regulation of clinical research. The protocol was approved by the Ethics Committee of the Brazilian National Cancer Institute (INCA #129/08), and all patients gave written consent to participate.

## Competing interests

The authors declare that they have no competing interests.

## Authors’ contributions

MSL recruited patients, collected clinical information, set the genotyping and expression assays, characterized genotypes and haplotypes, performed statistical analyses, generated tables and figures, and drafted the manuscript. LCG recruited patients, collected clinical information, and helped with genotyping assays. DNP set and performed the expression assays, and helped revising the manuscript. JSF-V collected and revised histopathological data, and helped revising the manuscript. VI-do-B recruited patients, collected clinical information, and helped with the statistical analyses. SK conceived the epidemiological design of the cohort. RSMN coordinated the genotyping of *(CA)n* polymorphism. MAC coordinated the expression assays, collaborated with data interpretation and revised the manuscript. RV-J conceived, designed, and coordinated the study, analyzed the data, wrote and revised the manuscript. All authors read and approved the final manuscript.

## Pre-publication history

The pre-publication history for this paper can be accessed here:

http://www.biomedcentral.com/1471-2407/14/190/prepub
